# Neck pain in South Africa: An overview of the prevalence, assessment and management for the contemporary clinician

**DOI:** 10.4102/sajp.v75i1.1332

**Published:** 2019-09-04

**Authors:** Cato A. Basson, Benita Olivier, Alison Rushton

**Affiliations:** 1 Department of Physiotherapy, University of the Witwatersrand, Johannesburg, South Africa; 2 Centre of Precision Rehabilitation for Spinal Pain (CPR Spine), School of Sport, Exercise and Rehabilitation Sciences, University of Birmingham, Edgbaston, United Kingdom

**Keywords:** neck pain, prevalence, assessment, management, risk factors, South Africa, physiotherapy

## Abstract

**Background:**

Neck pain is a prevalent condition and is associated with high levels of disability and pain. The long-term prognosis can be poor, and therefore effective management in the acute stage is important.

**Objectives:**

To provide an overview of the prevalence of neck pain and physiotherapy management and to provide evidence-informed recommendations for clinical practice within a South African context.

**Method:**

The literature was reviewed considering prevalence, risk factors and examination. Management recommendations were derived from the highest levels of evidence of clinical practice guidelines, systematic reviews and randomised clinical trials.

**Results:**

Neck pain is classified into four grades, and three trajectories of recovery have been identified. Although the incidence of neck pain globally is high, in the South African context the majority of the population have limited access to physiotherapy management. Sound clinical reasoning is important in the assessment and decision-making process for management. Exercise, and mobilisation or manipulation are effective treatment options in the management of most types of neck pain. Other physical modalities such as needling, transcutaneous electrical nerve stimulation, laser and intermittent traction may be used as an adjunct to management.

**Conclusion:**

The burden of neck pain globally is high; however, there is a lack of information on current practice, prevalence and burden of neck pain in the South African context. Sound evidence-informed clinical reasoning to inform a working diagnosis and to enable patient-centred management is important.

**Clinical implications:**

A thorough assessment is essential to gather information to formulate hypotheses regarding diagnosis and prognosis for neck pain. Exercise, and mobilisation or manipulation are effective management options.

## Introduction

Neck pain is one of the most common debilitating musculoskeletal complaints affecting the population (Hoy et al. 2014). In the 2015 Global Burden of Disease report, low back pain and neck pain were the leading causes of disability (Vos et al. 2016). Hoy et al. (2014:1309) defined neck pain as ‘pain in the neck with or without referred pain into one or both upper limbs’ and performed a systematic review to evaluate incidence, prevalence, duration and mortality risk of neck pain.

The considerable burden of disease from neck pain necessitates the effective translation of research findings into clinical practice. This article aims to give an overview of the prevalence of neck pain and physiotherapy management of neck pain and to make evidence-informed recommendations for application into clinical practice within a South African context.

## Prevalence and incidence of neck pain

From systematic review data, the prevalence of neck pain over a 12-month period was documented as 30% – 50% (Hogg-Johnson et al. 2008), with a point prevalence of 4.7% and a lifetime prevalence of 14.2% – 70% dependent on the country where it was measured (Hoy et al. 2014). The point prevalence of neck pain in the sub-Saharan Southern Africa region is high (males 4.7%; females 6.7%) and is only outranked by the United States (males 5.3%; females 7.6%), Western Europe (males 5.2%; females 7.4%) and East Asia (males 4.8%; females 7.0%) (Hoy et al. 2014; Vos et al. 2012). There was also a significant increase of neck pain prevalence between 1990 and 2015 (Hurwitz et al. 2018). Possible reasons cited for the increase in neck pain are an ageing population and increasing obesity (Hurwitz et al. 2018). From estimates for the global burden of neck pain (2000–2010), it was found that neck pain is more prevalent in women compared to men and peaks between 40 and 50 years of age (Hoy et al. 2014).

Few studies exist on the incidence of neck pain in the South African population (Brink et al. 2009; Mafanya & Rhoda 2011; Smith et al. 2009). Most of these only included an adolescent population. In a study of risk factors for developing neck pain amongst 181 adolescents in South Africa (SA), the incidence of neck pain was 53.7% (Mafanya & Rhoda 2011). Smith et al. (2009) reported a 20% incidence of neck pain in adolescent computer users (*n* = 1073), and similarly an incidence of 26% of neck pain was documented in a study on the sitting posture of South African adolescents (Brink et al. 2009). The incidence of musculoskeletal disorders of office workers in a private hospital in SA (Zungu & Ndaba 2009) was found to be 76% musculoskeletal complaints, with low back pain being the most common complaint followed by neck pain. In private physiotherapy practices in Pretoria, 46% of the musculoskeletal complaints seen by physiotherapists were patients with neck pain (Basson et al. 2017b).

## Burden of neck pain and access to health care in South Africa

Estimates of the burden of disease for SA in 2000 ranked musculoskeletal disorders 20th (Bradshaw et al. 2003), and the World Health Organization (2010) ranks it 16th from a more recent fact sheet on health statistics (1990–2008). In a primary care setting in SA, neck pain is ranked 34th as a main complaint for consultation (Mash et al. 2012). According to Rice, Smith and Blyth (2016), there is little information available for sub-Saharan Southern Africa in terms of the global burden of pain. The lack of data may be part of the reason for the large discrepancy in the ranking of musculoskeletal diseases in SA compared to the global burden of disease (Hoy et al. 2014; Vos et al. 2012).

The majority (84%) of the South African population use public health care. These facilities are often understaffed with limited resources (Ranchod et al. 2017). Prolonged waiting times in the public sector, mainly because of resource limitations, play a major role in the effective management of patients with neck pain (Hasumi & Jacobsen 2014). Access to public health care facilities depends on the area, with access often limited in rural areas because of distance from a facility and transport difficulties (National Department of Health 2015). Therefore, access to physiotherapy is also limited. There are 7698 physiotherapists registered in SA (Health Professions Council of South Africa 2018) of whom only 1258 are employed in public health care (National Department of Health 2015). Furthermore, of the 4595 physiotherapists who are members of the South African Society of Physiotherapists (SASP), 3671 work in the private sector (SASP data November 2017). Access to the private sector requires self-payment or membership of a medical insurance scheme. It could therefore be hypothesised that the majority of South African society will not have easy access to public health care facilities and physiotherapy management for neck pain. This may change when the National Health Insurance (NHI) is adopted (National Department of Health 2016) as it aims to make essential health care available to all South African citizens.

## Classification of neck pain

The Bone and Joint decade task force on neck pain and the Global Spine Care Initiative classify neck pain into four grades using a best evidence synthesis (Guzman et al. 2009; Haldeman et al. 2018) (S21 & S889).

Grade I – neck pain with no signs of major pathology and no or little interference with daily activities; Grade II – neck pain with no signs of major pathology, but interference with daily activities; Grade III – neck pain with neurologic signs of nerve compression; Grade IV – neck pain with signs of major pathology.

This classification system is also used in the recently updated Dutch clinical guidelines for neck pain (Bier et al. 2018). In contrast, the American Physical Therapist Association (APTA) clinical guidelines use a different classification system, namely neck pain with mobility difficulties; neck pain with movement control difficulties; neck pain with radiating pain; and neck pain with headache (Blanpied et al. 2017). For the purpose of this article, we will use the Global Spine Care Initiative definition (Haldeman et al. 2018) rather than the APTA classification (Blanpied et al. 2017), as it can be difficult to differentiate between mobility problems and control problems and they often overlap. We will also briefly discuss neck pain associated with headache and neck pain because of whiplash injury.

## Clinical course and risk factors for neck pain and recovery from neck pain

Three trajectories of recovery from neck pain have been identified (Van Hulst et al. 2016; Walton et al. 2014a). Rapid recovery over a month period is present in 19.6% of the neck pain population, 65.8% of patients have a modest rate of recovery (i.e. a non-significant decrease in pain and disability over a month period) and 14.6% have a pattern of worsening symptoms (Walton et al. 2014a). First onset neck pain tends to improve in the first 6 weeks after which symptoms often remain unchanged at 12-month follow-up (Hush et al. 2011; Vasseljen et al. 2013). Therefore, only around 20% of the neck pain population will recover well, and overall, the prognosis of acute neck pain is poor according to a systematic review and meta-analysis (Hush et al. 2011). At 12-month follow-up, the majority of patients still present with high pain severity and disability (Hush et al. 2011). A number of factors have high to moderate predictive power for the development of chronic pain (Walton et al. 2013).

Risk factors for first onset neck pain were found to be working in awkward positions (OR 1.65; 95%CI 1.04–2.60), sustained positions (OR 1.80; 95%CI 1.16–2.81) and psychosocial factors such as high job demands (RR 2.14; 95%CI 1.28–3.58) and co-worker support (RR 2.43; 95%CI 1.11–5.29) (Kim et al. 2018). Risk factors for poor prognosis from neck pain at baseline are high levels of pain (OR 5.61, 95%CI 3.74–8.43) and disability (NDI > 15/50; OR 42.18) at baseline (Walton et al. 2013). Other studies have highlighted the importance of psychosocial factors such as catastrophising (Karels et al. 2007; Thompson et al. 2010) and fear-avoidance beliefs (Karels et al. 2007; Pool et al. 2010) as risk factors for chronicity.

Social factors such as work–family imbalance and a hostile work environment have also been linked to a high prevalence of neck pain (Yang et al. 2016). In working populations, high job strain (OR 1.5 [95% CI 1.0 to 2.4]) and sleep disturbances (OR 2.2 [95% CI 1.6 to 3.0]) are associated with chronic neck pain (Rasmussen-Barr et al. 2014). Non-modifiable risk factors identified are being female and of older age (Walton et al. 2013). Longer duration of symptoms, absence of paraesthesia, a high neck pain intensity, disability at baseline and restricted range of movement (ROM) towards the affected side are found to be factors related to poor recovery of pain and disability (median under the curve 0.75–0.79) in cervical radiculopathy, a subgroup of neck pain (Sleijser‑Koehorst et al. 2018).

Neck pain is often associated with headache, upper back and shoulder or arm pain (Lindgren 2008; Salt et al. 2011). In a study of over 1800 patients, only 36% presented with neck pain alone and the remainder of the population had neck pain and radiating arm pain (Rasmussen et al. 2015). Radiating neck and arm pain and headache can have a negative impact on disability and quality of life (Daffner et al. 2003; De Pauw et al. 2015). Neuropathic pain is often present in patients with cervical radiculopathy (Tampin, Slater & Briffa 2013) and whiplash-associated disorders (WAD) (Sterling & Pedler 2009). On the website of the International Association for the Study of Pain, neuropathic pain is described as ‘pain initiated or caused by a primary lesion or dysfunction in the nervous system’ (International Association for the Study of Pain 2011). The presence of neuropathic pain has a negative impact on quality of life (Doth et al. 2010; Smart et al. 2012; Smith et al. 2007) and is also associated with disability and poor treatment outcomes (Blyth 2017; Doth et al. 2010). Table 1 summarises the risk factors for poor recovery reported by Jakobsen et al. (2018), Kim et al. (2018) and Walton et al. (2013).

**TABLE 1:** Predictors of poor recovery.

Risk factor	Confidence
High baseline pain	High
High baseline disability	High
Older age	Moderate
Psychosocial factors	Moderate
Working in awkward positions	Low
History of other musculoskeletal disorders (MSK) disorders	Low

## Assessment of patients with neck pain

**FIGURE 1:**
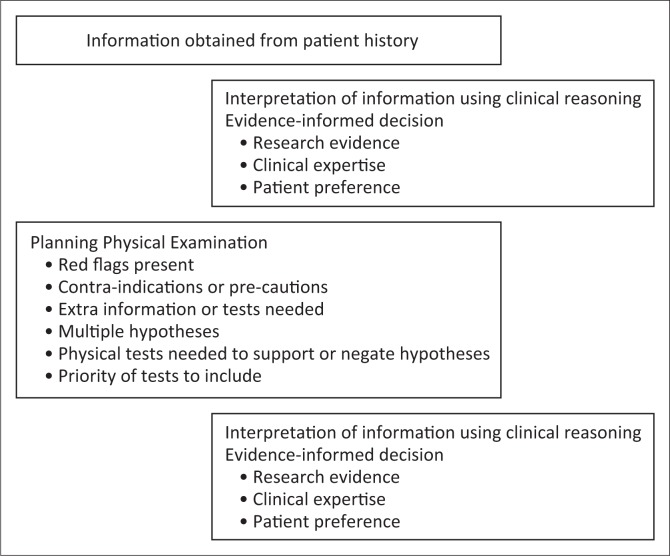
Planning the physical examination.

**FIGURE 2:**
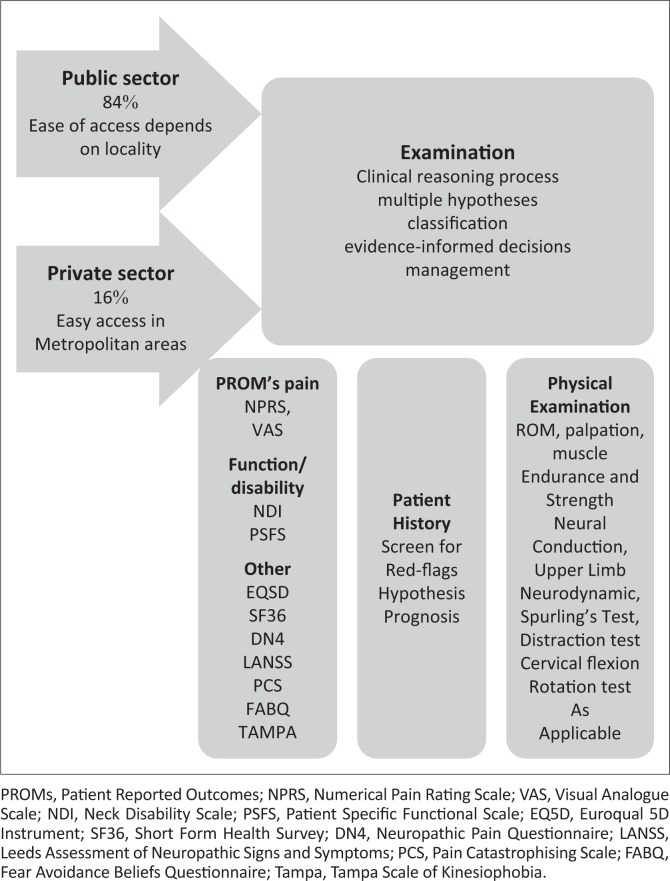
Patient examination.

Sound clinical reasoning is important to guide decision-making (Bier et al. 2018; Edwards et al. 2004; Rushton et al. 2014), and critical use of evidence should underpin the clinical reasoning process. According to Sackett et al. (1996), evidence-informed practice takes into account the best current research evidence, clinical expertise and patient preferences. Before deciding on management options, it is necessary to exclude serious pathology (Guzman et al. 2009) and refer patients with red flags for further investigation or to another health care practitioner. It is also important to determine whether the pain is acute (< 6 weeks since onset), sub-acute (6–12 weeks since onset) or chronic in nature (> 3 months duration). The treatment approach may differ between a patient with acute neck pain and one with chronic neck pain. The management of chronic pain is often more complex and can be treatment resistant (Cohen & Hooten 2017). Planning of the patient assessment is shown in Figure 1, while the various components that form part of the patient examination are shown in Figure 2.

### Patient history

The patient history is essential to gather information in order to formulate hypotheses regarding diagnosis and prognosis as well as the psychosocial context of the patient, and to identify the presence of possible red flags. The International Federation of Orthopaedic Manipulative Physical Therapists (IFOMPT) developed an international framework for the examination of the cervical spine for cervical arterial dysfunction (CAD) (Rushton et al. 2014). In the clinical reasoning process, examination for risk factors for CAD prior to using any manual therapy techniques on the cervical spine can assist to prevent adverse events (Hutting et al. 2018; Rushton et al. 2014). The physiotherapist should aim to identify factors that may potentially implicate neurovascular pathology or are indicative of contraindications to treatment (Rushton et al. 2014).

### Patient reported outcome measures

Patient reported outcome measures (PROMs) assist the clinician in establishing baseline characteristics such as pain, function, disability, quality of life and psychosocial factors. These measures also help to monitor the patient throughout the course of treatment enabling evaluation of treatment interventions and progression of interventions (Kuhn 2016). Additionally, it is proposed that the area of pain (body chart), behaviour of pain, pain intensity and quality of pain are documented (Fillingim et al. 2016). The multiple domains of pain (e.g. sensory, cognitive and affective) should also be assessed. This information can assist in the classification of the type of pain and possible underlying pathophysiological mechanisms (Fillingim et al. 2016).

The Numeric Pain Rating Scale and the Visual Analogue Scale are commonly used to measure pain. Both are reliable and sensitive to change (Holdgate et al. 2003). The Neck Disability Index and Patient Specific Functional Scale are recommended by current guidelines to measure function and disability (Bier et al. 2018; Blanpied et al. 2017). Other useful PROMs are the SF36 and EuroQual 5D Instrument to measure health-related quality of life (Coons et al. 2000). There is an array of instruments to assess psychosocial factors (Turk et al. 2016). The Fear-Avoidance Beliefs Questionnaire (Waddell et al. 1993) and the Tampa Scale for Kinesiophobia are valuable instruments used to assess the presence of fear-avoidance beliefs or kinesiophobia in the patient (Miller, Kori & Todd 1991). The Pain Catastrophising Scale can be used to assess the presence of high pain catastrophising (Sullivan, Bishop & Pivik 1995). The Diagnostic Pain Questionnaire (DN4) (Bouhassira et al. 2005) is recommended for use by the ‘South African Management of Neuropathic Pain Guidelines’ (Chetty et al. 2012:315) to assess the presence of neuropathic pain. The Leeds Assessment of Neuropathic pain, Signs and Symptoms (S-LANNS) is also advocated (Haanpää et al. 2011) to assess for the presence of neuropathic pain (Bennett 2001).

### Physical examination

Planning of the physical examination plays an important role in the effectiveness of the execution of the physical examination and is outlined in Figure 2. The physical examination enables testing of working hypotheses regarding diagnosis and prognosis. Active movement tests can be a valuable tool to establish a baseline and to monitor change over time (Blanpied et al. 2017). However, clinicians should not rely solely on ROM to make clinical decisions as other factors may play an important role in clinical presentation and prognosis (Snodgrass et al. 2014). Tools that are commonly used to measure the cervical ROM are the cervical range of motion (CROM) device (Performance Attainment Associates, Lindstrom, MN), the inclinometer and the standard goniometer (ICCs range between 0.89 and 0.98) (Audette et al. 2010; Snodgrass et al. 2014). Because of financial constraints in a SA context, use of the goniometer would be a common choice.

Passive accessory intervertebral movements of the cervical and thoracic spine can be useful, but reliability is poor to moderate except for assessing C1/C2 and C2/C3 levels (Hall et al. 2008). Reliability of the upper cervical mobility tests is better in symptomatic compared to asymptomatic participants (reliability fair: symptomatic range κ 0.21–0.40) (Van Trijffel et al. 2005).

Neck pain may be because of specific causes such as fracture, inflammatory disease and neurological compromise. However, for most of the neck pain population, an insidious onset is the cause of neck pain and therefore referred to as non-specific neck pain (Hoving et al. 2006).

Rubinstein et al. (2007) found that a cluster of tests aided in diagnosing cervical radiculopathy. These tests include neck rotation < 60°, a positive Spurling test, a positive Valsalva test and a positive neck distraction test. If three of the tests are positive, the probability of being a positive cervical radiculopathy was determined as 65% (6.1 [2.0–18.6]) (Wainner et al. 2003). A negative Upper Limb Neurodynamic Test (median nerve bias) would rule out cervical radiculopathy (Rubinstein et al. 2007).

The cervical flexion rotation test is a valid and reliable test to diagnose and evaluate cervicogenic headache (Hall et al. 2008; Rubio-Ochoa et al. 2016). Manual examination by means of palpation is a sensitive test to discriminate between cervicogenic headache and other headaches (Zito, Jull & Story 2006).

There is evidence that patients with radiating arm pain have sensory abnormalities (Moloney, Hall & Doody 2015). Therefore, it is advisable to perform neural conduction testing and, if the physiotherapist has access to an algometer, to test pressure pain threshold (Walton et al. 2014b).

## Management

The above process of examination enables a working hypothesis for diagnosis to enable the development of an individual patient management plan. Most guidelines recommend a combination of treatment modalities in the management of neck pain (Bier et al. 2018; Blanpied et al. 2017; Bussières et al. 2016; Childs et al. 2008; Cohen & Hooten 2017). The management options discussed below are derived from clinical practice guidelines (Bier et al. 2018; Blanpied et al. 2017), systematic reviews and randomised clinical trials. Table 2 outlines the levels of evidence used (Phillips et al. 2009).

**TABLE 2:** Levels of evidence.

Level	Type of evidence
1A	Systematic review (with homogeneity) of randomised controlled trials (RCTs)
1B	Individual RCT (with narrow confidence intervals)
1C	All or none study
2A	Systematic review (with homogeneity) of cohort studies
2B	Individual cohort study (including low-quality RCT, e.g. < 80% follow-up)
2C	‘Outcomes’ research; ecological studies
3A	Systematic review (with homogeneity) of case-control studies
3B	Individual case-control study
4	Case series (and poor quality cohort and case-control study)
5	Expert opinion

*Source*: Phillips, B., Ball, C., Sackett, D., Badenoch, D., Straus, S., Haynes, B. et al., 2009, *Oxford centre for evidence-based medicine – Levels of evidence*, 1998 edn., Centre of Evidence Based Medicine, Oxford

It is also important to differentiate between acute and chronic pain as this will determine the treatment approach (Grichnik & Ferrante 1991). Acute and sub-acute pain is described as the pain caused by an injury or disease and of recent onset. Chronic pain is pain that has been present for longer than 3 months and persists past normal tissue healing (Merskey & Bogduk 2011). In acute pain, treatment is aimed at the underlying cause, while treatment of chronic pain should be multimodal in nature (Grichnik & Ferrante 1991).

### Acute and sub-acute grades I and II neck pain

This describes neck pain with no signs of major pathology and either without (Grade I) or with (Grade II) interference with daily activities. These grades include non-specific neck and arm pain without any neurological compromise (Haldeman et al. 2018).

#### Mobilisation or manipulation

Level IA evidence supports mobilisation or manipulation for acute and sub-acute neck pain to improve pain and function in the short and medium term (Gross et al. 2015). This is in line with guideline recommendations (Bier et al. 2018; Blanpied et al. 2017). Thoracic manipulation can also be useful in the management of non-specific neck pain (Huisman, Speksnijder & De Wijer 2013) (Level IB). The decision regarding the choice between mobilisation and manipulation should be made after careful examination, clinical reasoning and assessment of risks and benefits.

#### Exercise

The evidence for exercises in acute neck pain is not robust, but the APTA guidelines recommend isometric strengthening and mobilising exercises (Blanpied et al. 2017). Mobilising exercises are effective for acute WAD (Teasell et al. 2010a). Strengthening exercises should be loaded progressively as aggressive strengthening exercises may aggravate pain during the healing phase (Teasell et al. 2010b) (Level 1A).

#### Advice to stay active

In patients with acute WAD (Teasell et al. 2010c) (Level 1A) and with acute neck pain (Bier et al. 2018) (Level 5), advice to remain active and continue with daily activities is recommended.

#### Other interventions

Other interventions that can be useful as an adjunct to mobilisation or manipulation and exercise are transcutaneous electrical nerve stimulation (TENS) (Kroeling et al. 2013) and dry needling (Mejuto-Vázquez et al. 2014) (Level 1B). The evidence does not support the use of a collar for acute neck pain as a collar may delay recovery in patients with acute WAD (Teasell et al. 2010c) (Level 1A).

Wirth, Humphreys and Peterson (2016) showed significant improvements in psychological parameters within the first month of acute and sub-acute neck pain. Therefore, if patients have not recovered as expected during the first month, psychosocial parameters should be re-evaluated and alternative management options explored.

### Chronic Grade I and II neck pain

#### Mobilisation or manipulation and exercise

Cervical and thoracic mobilisation are advocated to improve pain and function in the short and intermediate terms (Gross et al. 2015) (Level 1A). A recent systematic review found that cervical, shoulder and scapula-thoracic strengthening exercises are effective in reducing pain and function over the short, intermediate and long term in chronic neck pain (Gross et al. 2016) (Level 1A). Some studies suggest that the combination of exercise and mobilisation or manipulation has a better effect than either alone (Miller et al. 2010). However, a more recent review found that the combination of exercise and mobilisation or manipulation is not more effective than either alone (Fredin & Lorås 2017) (Level 1A). As the effect of exercise is evident over the long term (Gross et al. 2016) and mobilisation or manipulation has short-term, medium and long-term effects on pain and disability in chronic neck pain (Gross et al. 2015), a combination of the two seems like a feasible option. Another recent review found that general exercise does not improve long-term pain and disability in patients with WAD (Griffin, Leaver & Moloney 2017).

#### Other interventions

There is good evidence for the use of neuroscience education for chronic musculoskeletal pain (Louw et al. 2011) and emerging evidence for its use specifically for chronic neck pain (Malfliet et al. 2018) (Level 1B).

As is the case in acute or sub-acute neck pain, dry needling and TENS can be used as an adjunct in chronic neck pain (Level 1A). Acupuncture, intermittent traction and laser are better than placebo for chronic neck pain (Graham et al. 2013) (Level 1A). A guideline developed for chiropractors recommends multimodal treatment and advice for patients with chronic WAD (Bussières et al. 2016).

### Neck pain with radiating arm pain (Grade III)

Grade III neck pain refers to neck pain with neurological signs of nerve compression or irritation such as cervical radiculopathy (Haldeman et al. 2018). There is limited evidence for the optimal management of radiating arm pain (Salt et al. 2011; Thoomes et al. 2013). However, according to systematic reviews and guideline recommendations, some evidence exists for exercise, manual therapy, intermittent traction and low level laser (Blanpied et al. 2017; Graham et al. 2013; Salt et al. 2011). Neural mobilisation improves pain in radiating neck and arm pain but the impact of neural mobilisation on function and disability is conflicting (Basson et al. 2017a) (Level 1A). Neck-specific exercises may improve pain and function in patients with WAD and signs of neurological deficit (Landén Ludvigsson, Peterson & Peolsson 2018) (Level 2B).

### Neck pain with headache

For acute, sub-acute and chronic headache, the use of cervical mobilisation or manipulation with strengthening exercises is most effective in decreasing pain in patients with cervicogenic headache (Racicki et al. 2013) (Level 1A). This is also in line with the guideline recommendation (Blanpied et al. 2017). The C1/2 self-snag can be used as a home exercise to relieve pain in headache (Racicki et al. 2013) (Level 1B).

### Neuropathic pain

Neuropathic pain is common in chronic pain populations and nerve-related pain populations (Blyth 2017). There is limited evidence for the use of physiotherapy to manage neck pain associated with neuropathic pain (Day et al. 2014). A multimodal treatment approach incorporating postural education, scapular stabilisation, neural mobilisation exercises, manual therapy and stretching exercises improves pain, disability and grip strength in patients with peripheral neuropathic pain (Level 2B). There is experimental evidence that exercise can improve pain (Cooper, Kluding & Wright 2016); however, more research is needed on the conservative management of neuropathic pain.

## Implications for private sector and public sector

Physiotherapists in the private sector will be in a position to use different modalities taking into consideration patient preference and available evidence. However, in the public sector, where physiotherapists will often only see a patient once, the aim should be to provide exercises and information on self-management. For both the private and public sector, the ultimate aim should be for the patient to manage a chronic condition independently and to reduce the risk of recurrences in the case of an acute or sub-acute condition. Aligning the patient management approach to the South African context is crucial.

**FIGURE 3:**
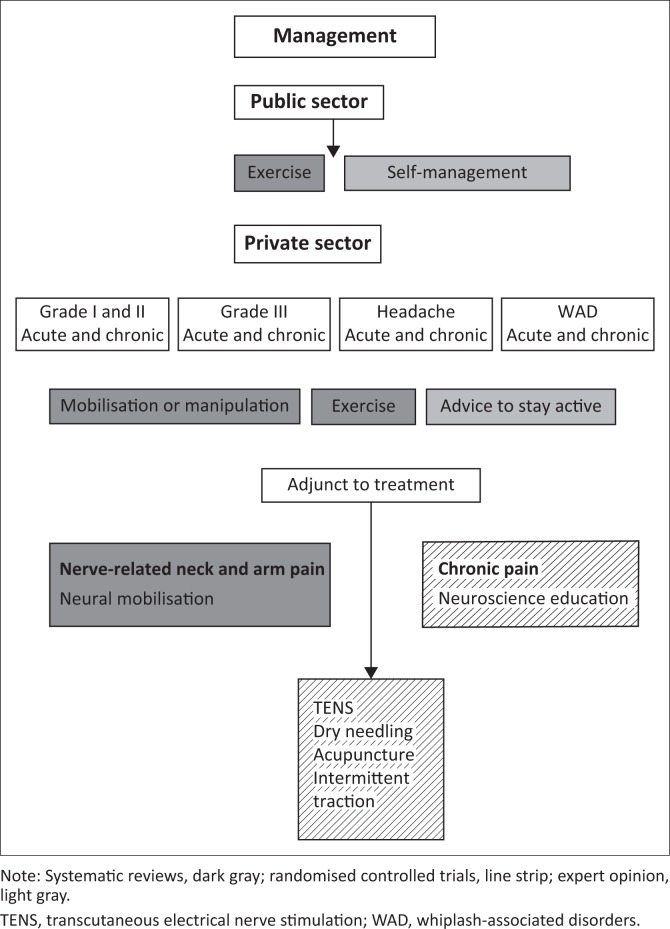
Management of neck pain.

South Africa has a unique multicultural landscape which calls for addressing the associated challenges surrounding communicative competence with regard to improving interviewing and counselling skills, intercultural and gender competencies, and linguistic and interpretation skills (Grant 2006). The management strategies to be considered for patients with neck pain are shown in Figure 3.

## Future directions

There is a dearth of information on neck pain prevalence and physiotherapy management in a South African context. There is a need for the development of culturally appropriate PROMs in different languages to assist in the assessment of patients. Risk factors for the development of neck pain and disability may differ in a multicultural environment (Geere et al. 2018) and should be further explored. Furthermore, the ideal management of neck pain in terms of patient outcomes and economic viability in a culturally diverse environment deserves urgent attention.

## Conclusion

Physiotherapists should use sound clinical reasoning to inform decisions pertaining to a working hypothesis and patient management in a patient-centred manner taking into account available evidence. The burden of neck pain globally is high; however, there is a lack of information on current practice, prevalence and burden in the South African context. The need for research on the prevalence and management of neck pain in SA is evident.

In summary:

- Neck pain is common and can be debilitating.
- Symptoms tend to improve over the first 6–8 weeks after which the symptoms will remain the same.
- The prognosis for full recovery at 1-year follow-up is poor.
- A comprehensive assessment and the use of outcome measures to monitor change over time are important.
- Strengthening exercise of the neck and scapula-thoracic area is of benefit to most types of neck pain.
- Mobilisation or manipulation improves pain over the short, intermediate and long term.
- Other physical modalities such as dry needling, TENS, laser and intermittent traction may be used as adjuncts to treatment.
